# Child development students’ perspectives on organic animal products: knowledge, attitudes and behaviors

**DOI:** 10.3389/fnut.2025.1619260

**Published:** 2025-06-25

**Authors:** Oğulcan Aral, Yusuf Cufadar, Gül Kadan, Neriman Aral, Burçin Aysu

**Affiliations:** ^1^Independent Researcher, ORSER, Ankara, Türkiye; ^2^Department of Animal science, Faculty of Agriculture, Selcuk University, Konya, Türkiye; ^3^Department of Child Development, Faculty of Health Sciences, Çankırı Karatekin University, Çankırı, Türkiye; ^4^Department of Child Development, Faculty of Health Sciences, Ankara University, Ankara, Türkiye; ^5^Department of Child Development, Faculty of Health Sciences, Ankara Yıldırım Beyazıt University, Ankara, Türkiye

**Keywords:** organic animal products, child development, university students, organic product, nutrition

## Abstract

Organic nutrition and its variant organic animal products constitute one of the most important areas of nutrition today. The consumption of organic animal products, which are known to make significant contributions to strengthening the immune system and ensuring a balanced and adequate diet, becomes even more important, especially for the development of children. For this reason, it is necessary to determine the extent of knowledge of child development specialist candidates, who work with children and families and have significant effects on children’s development, about organic animal products. Based on this idea, the aim of the research was to examine the knowledge, attitudes and behaviors of university students studying at universities that provide associate and undergraduate child development education in Türkiye through the screening method. The screening model, which is one of the quantitative research methods, was used in the study and it was conducted with the students of the child development department who continue their associate and undergraduate education in the child development program throughout Türkiye. The data were collected with the “Knowledge, Attitude and Behavior Survey Form for Organic Animal Products” developed by Aral and Çufadar. As a result of the study, it was determined that the scores of male students, those with a birth date of 2000 and below, associate degree students, those who know organic products, those who consume organic products, and those who look at the certificate when buying organic products were significantly higher in the behavioral dimension. In addition, in the attitude dimension, it was also found that the scores of those with a birth date of 2000 and below, those who consume organic products and have knowledge about organic products, those who look at the certificate when buying organic products and associate degree students were significantly higher. Moreover, it was concluded that there was a weak relationship between knowledge and behavior dimensions and attitude dimension. Based on the results obtained from the study, it can be suggested that students should be informed about organic animal products and necessary measures should be taken to facilitate access to these products.

## 1 Introduction

Organic agriculture is generally considered as practices that protect human health and contribute to the sustainability of the ecosystem in nature. In other words, organic agriculture is considered as practices that are based on the protection of animals or plants whose existence and future are in danger every day in nature, and that also have positive contributions to human health (1, 2). In this context, organic animal products in particular have a very important place. Organic animal products are considered as the raising of farm animals in their natural environments, where they are allowed to behave naturally, are allowed to be fed completely with organic products, and do not have synthetic products such as antibiotics in their feed. The most important feature of organic animal products that distinguishes them from other animal products in this context is that they are completely natural and are fed as they would be in their natural environments, without any residues or similar products that animals need (3). Moreover, this situation is important not only for the sustainability of nature, but also for the health of people. Unsaturated fatty acids found in organic animal products have the capacity to prevent many systemic or non-systemic diseases, especially obesity and cardiovascular problems (4). For this reason alone, it is preferred more.

Today, the interest and purchasing frequency of organic food and organic animal products, which are a type of these foods, are increasing. In fact, the demand for organic animal products, which are said to be healthy and should be a part of daily nutrition, is increasing day by day (1). Türkiye is a country with significant potential in terms of growing and consuming organic products. According to the data of the Ministry of Environment, Urbanization and Climate Change of the Republic of Turkey (2023), there has been a significant increase in the production of organic products in Turkey. It is stated that the number of production and consumption of organic products reached 1.5% in 2022 from 0.4% in 2002. This increase in the rate shows that the production and consumption of organic products is increasing day by day. (2). Especially in cases such as the pandemic experienced in the last century, which can cause mass deaths by affecting the immune system, people’s interest and demand for healthy nutrition and organic products have increased even more (3). At this point, the interest and attitudes of families toward their children’s nutrition can also be decisive.

The first introduction of families to organic products usually starts after their babies are born and this interest and desire continues for a lifetime. Families introduce their children to organic foods from a very early period for reasons such as healthy nutrition and resistance to diseases (4, 5). Especially in such periods when energy intake should be intense, families need to support the development of their children with foods that meet nutrients such as protein, carbohydrates and minerals in organic animal products (6). It is possible to explain this increase in the production and consumption of organic products by linking it to obesity and cardiovascular diseases, which are very important today. The unsaturated fatty acids found in organic animal products can have significant effects on the prevention of obesity and cardiovascular diseases. As a result of excessive nutrition with saturated fatty acids, LDL cholesterol increases and HDL cholesterol decreases. The decrease in HDL cholesterol can cause weight gain, weight gain can cause obesity or cardiovascular diseases (7, 8). However, in addition to their benefits, information on the negative effects of organic animal products on children’s development may cause confusion among families (8). In particular, the presence of pesticides in organic products or the information obtained on this subject can constitute a significant limitation in the consumption of organic products. The presence of pesticides in the materials used during the production of organic products increases the risk of contamination of these microorganisms (9–12). In particular, the presence of such microorganisms in organic products or the inability to find organic products that are completely beneficial to health can become a significant obstacle for consumers to access these products (9, 11, 13). Moreover, the increase in the prices of organic products that are beneficial to health in this context can become a significant obstacle in affecting the preferences of consumers in a country with a low economic level such as Turkey. As a matter of fact, studies have determined that pesticides are a significant obstacle to the consumption of organic products (9–11, 14, 15). Especially in milk and dairy products, which is an important organic animal product, the beliefs of families that there are antibiotic residues may push their children away from organic animal product consumption (16). However, the consumption of organic products, whose usefulness has been tested experimentally and which, contrary to popular belief, have significant effects on children’s development, should be increased (8, 17–19). Child development specialists have a great role in preventing such misinformation that may occur in families.

The child development department aims to train professionals who optimize the development of children between the ages of 0 and 18 who are at risk and who have typical and atypical development. Child development specialists, who make recommendations to families regarding the results obtained in the evaluation of children’s development, also provide consultancy to families in many areas, especially in the health and nutrition of their children (20, 21). Therefore, it is important that university students who are trained as child development specialists know organic animal products as well as the level of use of this knowledge.

In the literature review, it was determined that there are studies examining the perspectives of university students toward organic animal products (22–29). However, there are no studies reflecting the perspectives of university students in the child development department. In this context, it is important to take the opinions of prospective child development specialists studying in associate and undergraduate degree programs of child development about organic animal products to show the extent of their awareness. Consumption of organic animal products is particularly important in terms of protecting health and preventing certain diseases (8). Therefore, it is necessary to reveal the perspectives of child development specialists, who are strong professionals in the lives of children and families, toward organic animal products and their behaviors toward these products. Indeed, based on the results obtained, it will be possible to identify the deficiencies and take precautions accordingly. However, this information and behaviors may also be affected by certain sociodemographic characteristics. Therefore, the aim of the study is to determine whether the perspective toward organic animal products is affected by different sociodemographic characteristics specific to child development specialists.

## 2 Limitations of the research

The research is an original article. It was carried out using the observational method to determine the opinions of university students toward organic products. The high number of female students in the study, the prejudices of the students toward their answers and the research model are among the important limitations. The main reason for the high number of females is that Child Development associate and undergraduate students are preferred more by female students (30). Therefore, although the number of female participants is higher, considering the necessity of addressing the information of all child development department students within the scope of the research purpose, it was found important to take the attitudes of male students into consideration and they were included in the study. In order for the students not to experience any influence, the students were informed in advance that all information would remain confidential. A cross-sectional study was preferred in the study to determine the knowledge and attitudes of the students. In further studies, causal comparisons should be made to determine the factors in determining the students’ organic product preferences.

## 3 Methods

The research model, study group, data collection tools, data collection method and data analysis sections are given below. The study was conducted with Child Development students who receive formal education in Türkiye. The study group consisted of students studying at the associate and undergraduate levels.

### 3.1 Research model

The survey model, one of the quantitative research methods, was used in the study. The survey model is a technique that requires data collection from a large sample group with valid and reliable measurement tools about any subject (31). In this context, the survey model was used to determine the opinions of students attending the associate and undergraduate programs of the child development department about organic animal products.

### 3.2 Study group

It was aimed to study with a large sample in order to determine the knowledge and attitudes of child development associate and undergraduate students in Turkey toward organic products. In this context, G*Power 3.1.9.7 was used. In this context, sample calculation was made for the independent groups *t*-test in the research. In the calculation, the effect size was low (d = 0.10), 5% margin of error (*α* = 0.05) and 95% power (1-*β* = 0.95) was taken and the sample number was determined as 1,073. With the thought that there might be data loss in the research, the number was increased by 5% and determined as 1,180 people. A survey was applied to 1,180 students in the research. However, this number was determined as 1,120 after the data losses. The sociodemographic characteristics of the students participating in the study are given in Table 1.

**Table 1:** Distribution of sociodemographic characteristics of university students.

Features	f	%
Gender		
Female	1,064	95
Male	56	5
Age		
2000 and below	134	12
2001–2003	667	59.6
2004 and above	319	28.5
Number of siblings		
Single	43	3.8
2–3	711	63.5
3 and above	366	32.7
Birth order		
First	450	40.2
Middle	388	34.6
Last	283	25.3
Education level		
Associate degree	492	43.9
Bachelor’s degree	628	56.1
Place of stay		
Home	280	25
Dorm	840	75
Organic product information		
Yes	91	8.1
No	1,029	91.9
Organic product consumption		
Not using	22	2
Sometimes	433	38.7
Using	665	59.4
Consumed product		
Milk and dairy products	880	22.6
Eggs	215	19.2
Red and white meat	18	1.6
Other	7	0.6
Reason for consumption		
Health benefits	399	35.6
Nutritional value	296	26.4
Safe	255	22.8
Environmentally friendly	144	12.9
Price	26	2.2
Transportation route		
Hometown	306	27.3
Family manufacturers	188	16.8
Local product market	166	14.8
Neighborhood market	158	14.1
Shopping center	136	12.1
Family	132	11.8
Internet	30	2.7
Other	4	0.3
Certificate check		
Do not know	385	34.4
No	446	39.8
Yes	289	25.8
Frequency of consumption		
Once a week	640	57.1
Once a month	170	15.2
Two or three times a month	150	13.4
Less frequency	96	8.6
Every day	64	5.7
Problems encountered		
Price	299	26.7
Hygiene	217	19.4
Lack of confidence	212	18.9
Appearance	104	9.3
Packaging	92	8.2
Certificate	57	5.1
Market share	50	4.5
Promotion	49	4.4
Shipping	34	3
No difficulty	6	0.4
Thought toward organic products		
Healthy	290	25.9
Delicious	206	18.4
Quality	163	14.6
Additive-free	146	13
Expensive	130	11.6
Environmentally friendly	109	9.7
Long shelf life	54	4.8
Nice looking	22	1.9
Source of organic product information		
Social media-internet	571	51
Family-relatives	510	45.5
Environment-university	39	3.5

G^*^3.1.7. Protocol. *t* tests, Correlation: Point biserial model. Analysis: A priori: Compute required sample size. Input: Tail(s) = One; Effect size |ρ| = 0.1; α err prob = 0.05; Power (1-β err prob) = 0.95; Output: Noncentrality parameter δ = 3.2921701; Critical t = 1.6462776; Df = 1,071; Total sample size = 1,073; Actual power = 0.9500394.

Of the students in the study, 95% were female, 5% were male, 59.6% were in the 2001–2003 age group, 28.5% were in the 2004 and above age group, and 12% were in the 2000 and below age group. 63.5% of the students had 2–3 siblings, 32.7% had 3 or more siblings and 3.8% were only children. 40.2% were born in the first row, 34.6% in the middle row and 25.3% in the last row. 56.1% attend undergraduate and 43.9% associate degree programs. 75% live in dormitories and 25% live at home. 66.9% have some knowledge about organic products, 25% know about organic products and 8.1% have no knowledge about organic products. 59.4% consume organic products, 38.7% use them occasionally and 2% do not use them. 22.6% of the students eat milk and dairy products, 19.2% eggs, 1.6% red and white meat, and 0.6% other (salami, sausage, fruit, etc.). 35.6% of the students consume organic animal products because they are beneficial for health, 26.4% because of their nutritional value, 22.8% because they are safe, 12.9% because they are environmentally friendly and 2.2% because of their price. Organic animal products are obtained by 27.3% of the students from their hometowns, 16.8% through familiar producers, 14.8% from local product markets, 14.1% from neighborhood markets, 12.1% from shopping centers, 11.8% through their families, 2.7% through the internet, and 0.3% through other means (from abroad). While 39.8% of the students do not look at the organic product certificate, 25.8% look at the certificate, and 34.4% do not know the organic certificate. Organic animal products are consumed once a week by 57.1% of the students, once a month by 15.2%, two to three times a month by 13.4%, less frequently by 8.6%, and every day by 5.7%. While 26.7% of students have problems with prices, 19.4% with hygiene, 18.9% with lack of trust, 9.3% with appearance, 8.2% with packaging, 5.1% with certification, 4.5% with market share, 4.4% with promotion, 3% with cargo, 0.4% have no difficulties. 25.9% of the students think that organic products are healthy, 18.4% think that they are delicious, 14.6% think that they are high quality, 13% think that they are additive-free, 11.6% think that they are expensive, 9.7% think that they are eco-friendly, 4.8% think that they have a long shelf life, and 1.9% think that they are nice looking. 51% of the students learn about organic products through social media-internet, 45.5% through family-relatives, and 3.5% through the environment-university.

### 3.3 Data collection tools

Data were collected with the “General Information Form” developed by the researchers and the “Knowledge, Attitude and Behavior Survey Form Toward Organic Animal Products” developed by Aral and Çufadar (32).

General Information Form: It was developed by the researchers in order to determine the sociodemographic characteristics of the university students in the study and their knowledge about organic products. The form includes information on students’ gender, date of birth, number of siblings, birth order, type of program they study, place of residence, whether they look at the certificate when buying organic products, which organic products they prefer, the reasons for preferring organic products, how often they consume organic products, the difficulties they experience in accessing organic products, their thoughts about organic products and where they get information about organic products.

Knowledge, Attitude and Behavior Survey Form Toward Organic Animal Products: This form was developed by Aral and Çufadar (32) to determine the knowledge, attitudes and behaviors of university students toward organic animal products. The knowledge form for organic animal products consists of 9 items, one dimension and a 2-point Likert type. The attitude form toward organic animal products consists of 23 items, 4 sub-dimensions (market, production, human and animal) and a 5-point Likert type, while the behavior form consists of 16 items, one sub-dimension and a 5-point Likert type. During the development process of the measurement tool, Cronbach’s Alpha values were determined as 0.68 for the knowledge form, 0.80 for the Market sub-dimension of the Attitude form, 0.78 for the Production sub-dimension, 0.79 for the Human sub-dimension, 0.74 for the Animal sub-dimension and 0.94 for the Behavior form. Within the scope of the study, Cronbach’s Alpha values were determined as 0.71 for the Knowledge Form, 0.84 for the Market sub-dimension, 0.80 for the Production sub-dimension, 0.81 for the Human sub-dimension, 0.88 for the Animal sub-dimension of the Attitude Form and 0.95 for the Behavior Form.

### 3.4 Data collection method

Firstly, ethics committee permission was obtained from Selçuk University in Türkiye, Faculty of Agriculture Scientific Ethics Evaluation Board with decision number 573405 dated 23.08.2023 in order to collect the data in the study. After the ethics committee permission was obtained, the associate and undergraduate child development departments in Türkiye were contacted. The lecturers were informed about the purpose of the study. In the study, university students were reached through faculty members and information about the study was provided. Students who wanted to participate in the study first filled out voluntary consent forms on the “Google Form,” and the names of the students were not taken. The students answered the research questions during a period when they were outside the school environment. Necessary explanations were made to the students in this regard.

### 3.5 Data analysis

SPSS IBM statistical program was used to analyze the data. The answers given by the students through “Google Forms” were transferred to the SPSS program. Descriptive analyses were made about the sociodemographic characteristics of the students and their knowledge about organic products. Kolmogorov Smirnov test results and skewness and kurtosis values were used to determine whether the answers given by the students to the survey forms of knowledge, attitude and behavior toward organic animal products were normally distributed or not since the number of samples was over 50.

Table 2 shows the results of the normality analysis test for the survey form of knowledge, attitudes and behaviors toward organic animal products of students studying in associate and undergraduate child development programs. In the literature, it is stated that skewness and kurtosis values are effective in determining whether the data show normal distribution (33) and these values between +2 and −2 are evidence for normal distribution (34). In this context, it can be stated that the data are normally distributed. In the analysis of the data in the study, independent samples t-test, ANOVA and Pearson Correlation Analysis test results were examined from parametric techniques.

**Table 2:** Normality analysis test results for the knowledge, attitude and behavior survey form toward organic animal products.

Scale	n	X	Skewness	Kurtosis	*p*
Knowledge	1,120	17.66	0.07	0.10	0.00
Market	1,120	26.95	−1.73	0.15	0.00
Production	1,120	26.74	−1.47	2.05	0.00
Human	1,120	22.34	−1.60	2.96	0.00
Animal	1,120	27.31	−1.98	0.45	0.00
Behavior	1,120	50.17	0.33	−0.62	0.00

## 4 Findings

The findings of the study conducted to examine the opinions of students studying in associate and undergraduate child development departments of universities toward organic animal products are given below.

Table 3 shows the means and independent sample *t*-test results of the knowledge, attitude and behavior survey form toward organic animal products according to the gender of the students studying in associate and undergraduate child development programs. As seen in the table, a significant difference was found in the behavior form of the knowledge, attitude and behavior survey form for organic animal products according to the gender of the students [t(1118) = −2.56; *p* < 0.005]. Accordingly, the arithmetic mean of male students (x = 55.18) is higher than the arithmetic mean of female students (x = 49.91).

**Table 3:** Means and independent samples *t*-test results of the knowledge, attitude and behavior survey form toward organic animal products according to the gender of the students studying in associate and undergraduate child development programs (*n* = 1,120).

Survey form	Gender	n	x̄	ss	sd	*t*	*p*
Knowledge	Female	1,064	17.66	0.94	1,118	0.16	0.87
	Male	56	17.64	0.82			
Market	Female	1,064	26.93	3.45	1,118	−0.88	0.38
	Male	59	27.30	3.04			
Production	Female	1,064	26.71	3.45	1,118	−1.18	0.24
	Male	56	27.27	3.39			
Human	Female	1,064	22.33	3.00	1,118	−0.59	0.53
	Male	56	22.57	2.78			
Animal	Female	1,064	27.29	3.44	1,118	−0.98	0.33
	Male	56	27.70	2.99			
Behavior	Female	1,064	49.91	1.80	1,118	−2.56	0.01
	Male	56	55.18	1.82			

*p* < 0.001.

Table 4 shows the means and ANOVA test results of the Knowledge, Attitude and Behavior Survey Form for Organic Animal Products according to the birth dates of the students studying in associate and undergraduate child development programs. As seen in the table, a significant difference was found in the market sub-dimension (*F* = 4.06; *p* < 0.005), human sub-dimension (*F* = 5.17; *p* < 0.005) of the attitude form and in the behavior form (*F* = 12.89; *p* < 0.001) of the knowledge, attitude and behavior survey form for organic animal products. In the market sub-dimension, the mean rank of those with a birth date of 2000 and below (x = 27.65) is higher than the mean rank of those with a birth date of 2004 and above (x = 26.54). In the human sub-dimension, the mean rank (x = 23.12) of those with birth dates 2000 and below is higher than the mean rank (x = 22.25) of those with birth dates 2001–2003 and higher than the mean rank (x = 22.22) of those with birth dates 2004 and above. In the behavior form, the mean rank of the students with a birth date of 2000 and below (x = 55.92) is higher than the mean rank of the students with a birth date of 2001–2003 (x = 49.96) and the mean rank of the students with a birth date of 2004 and above (x = 48.20).

**Table 4:** Means and ANOVA test results of the knowledge, attitude and behavior survey form toward organic animal products according to the date of birth of the students studying in associate and undergraduate child development programs (*n* = 1,120).

Survey form	Age	n	x̄	ss	Sum of squares	sd	F	*p*	Significant difference
Knowledge	2000 and below^1^	134	17.57	1.01	1.89	2	1.18	0.31	
	2001–2003^2^	666	17.69	0.90	890,85	1,117			
	2004 and above^3^	320	17.65	0.92	892,74	1,119			
Market	2000 and below^1^	134	27.65	0.98	95.10	2	4.06	0.02	1–3
	2001–2003^2^	666	26.96	0.13	13,067,39	1,117			
	2004 and above^3^	320	26.64	0.19	13,162,49	1,119			
Production	2000 and below^1^	134	27.28	0.30	51.09	2	2.16	0.12	
	2001–2003^2^	666	26.73	0.14	13,222,30	1,117			
	2004 and above^3^	320	26.54	0.19	13,273,39	1,119			
Human	2000 and below^1^	134	23.12	0.25	91.80	2	5.17	0.01	1–2
	2001–2003^2^	666	22.25	0.12	9,908,85	1,117			1–3
	2004 and above^3^	320	22.22	0.16	10,000,66	1,119			
Animal	2000 and below^1^	134	27.79	0.29	51.41	2	2.20	0.11	
	2001–2003^2^	666	27.33	0.13	13,024,46	1,117			
	2004 and above^3^	320	27.06	0.18	13,075,87	1,119			
Behavior	2000 and below^1^	134	55.92	1.31	5,996,60	2	12.89	0.00	1–2
	2001–2003^2^	666	49.96	0.56	246,819,79	1,117			1–3
	2004 and above^3^	320	48.20	0.87	252,516,40	1,119			

*p* < 0.05. Group 1 represents university students aged 2000 and below group 2 represents university students aged between 2001-2004, group 3 represents university students aged 2004 and above.

Table 5 shows the means and independent sample t-test results of the Knowledge, Attitude and Behavior Survey Form for Organic Animal Products according to the program type of the students studying in associate and undergraduate child development programs. As can be seen in the table, a significant difference was found in the production sub-dimension [t(1118) = 0.78; *p* < 0.005], human sub-dimension [t(1118) = 0.33; *p* < 0.001], animal sub-dimension [t(1118) = 2.37; *p* < 0.005] and behavior form [t(1118) = 4.10; *p* < 0.001]. In the production sub-dimension, the mean rank of the students studying in the associate degree program (x = 27.02) is higher than the mean rank of the students in the undergraduate program (x = 26.52). In the People sub-dimension, the mean rank of the students in the associate degree program (x = 22.73) is higher than the mean rank of the students in the undergraduate program (x = 22.04). In the animal sub-dimension, the mean rank of the students in the associate degree program (x = 27.58) is higher than the mean rank of the students in the undergraduate program (x = 27.10). In the behavior form, the mean rank of the students in the associate degree program (x = 52.24) is higher than the mean rank of the students in the undergraduate program (x = 48.55).

**Table 5:** Means and independent sample *t* test results of knowledge, attitude and behavior survey forms for organic animal products of students studying in associate and undergraduate child development programs (*n* = 1,120).

Survey form	Type of program	n	x̄	ss	sd	*t*	*p*
Knowledge	Associate Undergraduate	492 628	17.66 17.68	0.78 0.97	1,118	−0.39	0.69
Market	Associate Undergraduate	492 628	27.17 26.78	3.24 3.56	1,118	0.16	0.06
Production	Associate Undergraduate	492 628	27.02 26.52	3.32 3.52	1,118	0.78	0.01
Human	Associate Undergraduate	492 628	22.73 22.04	2.82 3.09	1,118	0.33	0.00
Animal	Associate Undergraduate	492 628	27.58 27.10	3.15 3.60	1,118	2.37	0.02
Behavior	Associate Undergraduate	492 628	52.24 48.55	4.37 5.55	1,118	4.10	0.00

*p* < 0.05.

Table 6 shows the means and ANOVA test results for the knowledge, attitude and behavior survey form according to the knowledge of the students studying in associate and undergraduate child development programs about organic animal products. Significant differences were found in the attitude form, human sub-dimension (*F* = 6.83; *p* < 0.001) and behavior form (*F* = 28.30; *p* < 0.001). In the human sub-dimension, the mean rank of the students with knowledge about organic products (x = 22.82) is higher than the mean rank of the students who have some knowledge about organic products (x = 22.26) and the students without knowledge about organic products (x = 21.59). In the behavior form, the mean rank (x = 55.75) of the students with knowledge about organic products is higher than the mean rank (x = 48.59) of the students who have some knowledge about organic products and higher than the mean rank (x = 45.99) of the students without knowledge about organic products.

**Table 6:** Means and ANOVA test results of knowledge, attitude and behavior survey form according to the knowledge of organic animal products of students studying in associate and undergraduate child development programs (*n* = 1,120).

Survey form	Organic product knowledge	n	x̄	ss	Sum of squares	sd	F	*p*	Significant difference
Knowledge	No^1^	91	17.59	0.92	1.55	2	0.97	0.38	
	Some^2^	749	17.66	0.97	891,19	1,117			
	Yes^3^	280	17.73	0.61	892,74	1,119			
Market	No^1^	91	26.50	4.08	69.05	2	2.94	0.05	
	Some^2^	749	26.86	3.36	13,093,45	1,117			
	Yes^3^	280	27.35	3.35	13,162,49	1,119			
Production	No^1^	91	26.44	3.80	42.19	2	1.78	0.17	
	Some^2^	749	26.66	3.34	13,231,20	1,117			
	Yes^3^	280	27.06	3.57	13,273,39	1,119			
Human	No^1^	91	21.59	3.71	120,85	2	6.83	0.00	1–3
	Some^2^	749	22.26	2.88	9,879,81	1,117			2–3
	Yes^3^	280	22.82	2.95	10,000,66	1,119			
Animal	No^1^	91	27.11	3.96	66,68	2	2.86	0.05	
	Some^2^	749	27.18	3.32	13,009,19	1,117			
	Yes^3^	280	27.73	3.46	13,075,87	1,119			
Behavior	No^1^	91	45.99	6.47	12,176,99	2	28.30	0.00	1–3
	Some^2^	749	48.59	4.59	240,339,41	1,117			2–3
	Yes^3^	280	55.75	4.26	252,516,40	1,119			

*p* < 0.05. Group 1 represents university students aged 2000 and below group 2 represents university students aged between 2001-2004, group 3 represents university students aged 2004 and above.

Table 7 shows the means and ANOVA test results for the knowledge, attitude and behavior survey form of the opinions of the students studying in associate and undergraduate child development programs toward the consumption of organic animal products. As seen in the table, significant differences were found in the production sub-dimension of the attitude form (*F* = 4.69; *p* < 0.005); in the people sub-dimension (*F* = 5.08; *p* < 0.005); and in the behavior form (*F* = 18.62; *p* < 0.001). In the production sub-dimension, the mean rank of the students who consume organic animal products (x = 26.96) is higher than the mean rank of the students who consume them occasionally (x = 26.35). In the human sub-dimension, the mean rank of the students who consume organic products (x = 22.56) is higher than the mean rank of the students who consume organic products occasionally (x = 21.99). In the behavior form, the mean rank of the students who consume organic animal products (x = 52.39) is higher than the mean rank of the students who consume organic animal products occasionally (x = 46.83).

**Table 7:** Means and ANOVA test results of knowledge, attitude and behavior survey form according to the consumption of organic animal products of students studying in associate and undergraduate child development programs (*n* = 1,120).

Survey form	Organic product consumption	n	x̄	ss	Sum of squares	sd	F	*p*	Significant difference
Knowledge	No^1^	22	17.82	0,66	2.65	2	1.66	0.19	
	Occasionally^2^	433	17.61	1.05	890,09	1,117			
	Yes^3^	665	17.70	0.78	892,74	1,119			
Market	No^1^	22	27.73	2.57	37.74	2	1.61	0.20	
	Occasionally^2^	433	26.75	3.44	13,124,75	1,117			
	Yes^3^	665	27.06	3.44	13,162,49	1,119			
Production	No^1^	22	27.50	2.82	110,46	2	4.69	0.01	2–3
	Occasionally^2^	433	26.35	3.67	13,162,93	1,117			
	Yes^3^	665	26.96	3.29	13,273,39	1,119			
Human	No^1^	22	22.77	2.88	90.07	2	5.08	0.01	2–3
	Occasionally^2^	433	21.99	3.07	9,910,59	1,117			
	Yes^3^	665	22.56	2.92	10,000,66	1,119			
Animal	No^1^	22	27.36	3.26	48.82	2	2.09	0.12	
	Occasionally^2^	433	27.05	3.49	13,027,05	1,117			
	Yes^3^	665	27.48	3.37	13,075,87	1,119			
Behavior	No^1^	22	48.86	9.99	8,148,55	2	18.62	0.00	2–3
	Occasionally^2^	433	46.83	4.36	244,367,85	1,117			
	Yes^3^	665	52.39	4.87	252,516,40	1,119			

*p <* 0.05. Group 1 represents university students who do not consume organic animal products, group 2 represents university students who occasionally consume organic animal products, and group 3 represents university students who consume organic animal products.

Table 8 shows whether the students studying in associate and undergraduate child development programs have knowledge about organic product certification and the averages and ANOVA test results of the organic animal product knowledge, attitude and behavior survey form. As seen in the table, significant differences were found in the market sub-dimension (*F* = 3.73; *p* < 0.005), production sub-dimension (*F* = 3.17; *p* < 0.005), people sub-dimension (*F* = 5.60; *p* < 0.001) of the attitude form and in the behavior form (*F* = 43.12; *p* < 0.001). In the market sub-dimension, the mean rank (x = 27.27) of the students with knowledge about the certificate is higher than the mean rank (x = 26.58) of the students without knowledge about the certificate. In the production sub-dimension, the mean rank of the students with certificate knowledge (x = 27.06) is higher than the mean rank of the students without certificate knowledge (x = 26.41). In the human sub-dimension, the mean rank of the students with certificate knowledge (x = 22.84) is higher than the mean rank of the students without certificate knowledge (x = 22.09) and the mean rank of the students who have certificate knowledge but do not look at the certificate when buying products (x = 22.24). In the behavior form, the mean rank (x = 56.93) of the students who with certificate knowledge is higher than the mean rank (x = 48.50) of the students without certificate knowledge and the mean rank (x = 47.24) of the students who have certificate knowledge but do not look at it.

**Table 8:** Means and ANOVA test results of knowledge, attitude and behavior survey form according to knowledge about the certificate of organic animal product of the students studying in associate and undergraduate child development programs (*n* = 1,120).

Survey form	Organic product certificate	n	x̄	ss	Sum of squares	sd	F	*p*	Significant difference
Knowledge	No^1^	446	17.61	1.11	4.39	2	2.76	0.06	
	Do not Know^2^	385	17.67	0.78	888,35	1,117			
	Yes^3^	289	17.77	0.59	892,74	1,119			
Market	No^1^	446	27.06	3.26	87.34	2	3.73	0.02	2–3
	Do not Know^2^	385	26.58	3.43	13,075,15	1,117			
	Yes^3^	289	27.27	3.64	13,162,49	1,119			
Production	No^1^	446	26.81	3.34	74.93	2	3.17	0.04	2–3
	Do not Know^2^	385	26.41	3.38	13,198,46	1,117			
	Yes^3^	289	27.06	3.65	13,273,39	1,119			
Human	No^1^	446	22.24	3.03	99,34	2	5.60	0.00	1–3
	Do not Know^2^	385	22.09	2.89	9,901,32	1,117			2–3
	Yes^3^	289	22.84	3.00	10,000,66	1,119			
Animal	No^1^	446	27.38	3.32	43.76	2	1.88	0.15	
	Do not Know^2^	385	27.05	3.38	13,032,11	1,117			
	Yes^3^	289	27.54	3.61	13,075,87	1,119			
Behavior	No^1^	446	47.24	5.02	18,099,87	2	43.12	0.00	1–3
	Do not Know^2^	385	48.50	3.97	234,416,52	1,117			2–3
	Yes^3^	289	56.93	4.32	252,516,40	1,119			

*p* < 0.05. Group 1 includes university students who do not know about the certification information for organic animal products, Group 2 includes university students who do not have information on this subject, Group 3 includes university students who know that there should be a certificate for organic animal products.

Table 9 shows the results of Pearson correlation analysis of the organic animal product knowledge, attitude and behavior survey form. As can be seen in the table, there is a low-level positive significant relationship between the knowledge form and the market (*r* = 0.07; *p* < 0.05), production (*r* = 0.07; *p* < 0.05) and animal (*r* = 0.08; *p* < 0.05) sub-dimensions. In addition, a weak positive correlation was found between the behavior form and the market (*r* = 0.28; *p* < 0.001), production (*r* = 0.31; *p* < 0.001), human (*r* = 0.37; *p* < 0.001) and animal (*r* = 0.29; *p* < 0.001) sub-dimensions.

**Table 9:** Pearson correlation analysis results of organic animal product knowledge, attitude and behavior survey form (*n* = 1,120).

Sub-dimensions	Market sub-dimension	Production sub-dimension	Human sub-dimension	Animal sub-dimension
Knowledge Sub-dimension	*r* = 0.07	*r* = 0.07	*r* = 0.05	*r* = 08
	*p* < 0.05	*p* < 0.05	*p* > 0.05	*p* < 0.05
Behavior Sub-dimension	*r* = 0.28	*r* = 0.31	*r* = 0.37	*r* = 0.29
	*p* < 0.001	*p* < 0.001	*p* < 0.001	*p* < 0.001

## 5 Discussion and conclusion

As a result of the research, a significant difference was found in the behavior dimension toward organic animal products according to the gender of the students. Organic products are generally among the products with high nutritional and quality values along with the price ratio. The high nutritional value of the product along with the price may result in shoppers purchasing these products in proportion to their financial situation (35, 36). In other words, in developing countries such as Turkey, it is seen that men, who work more to meet the economic situation at home, prefer organic products more (37–39). As a result of the research, it is noteworthy that male students received higher scores in this context. The findings of the other studies that gender is a determinant in organic product consumption confirm the results found in this study (40–45).

As a result of the research, it is noteworthy that the tendency toward organic products increases with increasing age. As it is known, the possibility of some health problems also increases with increasing age. Studies conducted on the benefits of organic products in preventing health problems that may arise, especially in cardiovascular diseases, can also lead to consumers acting with this awareness (36, 46–50). As a result of the research, it is seen that university students’ behaviors toward organic products are more positive as their age increases. Thus, it is thought that it can be effective in preventing many health problems, especially cardiovascular diseases (46, 51–54), and they reflect this situation in their behavior. When considered in this context, as the age of university students increases, it may bring the potential to bring along more attitudes and behaviors toward organic animal products (32). As a matter of fact, the results obtained in the studies confirm our finding that the consumption of organic products increases in parallel with the increase in age (27, 47, 55, 56).

The results of the study show that students attending the associate degree program in child development have a more positive attitude and behavior toward organic animal products. Particularly, the fact that the majority of students continuing their associate degree education live with their families, as well as the lack of confidence that students frequently express about organic products and factors such as price can prevent undergraduate students living away from their families from accessing these products (28, 57–60). In Turkey, especially as a developing country and due to low economic income, expenditure on basic nutrients can also be affected in this context. Indeed, the high prices of organic products in particular can have significant effects on consumers’ purchasing preferences, and therefore the purchasing level decreases (23, 27). However, at the same time, the fact that Turkey is an agricultural country can also help the environment to consume organic products at least at certain times (58–61). It is seen that these situations are reflected in the results.

Another finding reached in the study is that students who have knowledge about organic products and look at the certificate when buying organic products exhibit more positive characteristics in terms of attitude and behavior. Organic products are used in many ways today, and sometimes there may be misconceptions. Especially the idea that there is more harm than benefit, which is spoken among the public, can create confusion in students who have no knowledge about organic animal products and may result in students staying away from these products. In addition, it is an important feature that organic products have a certificate, which is a promotional card. Unconscious production, which can be a great danger in the organic animal food market, can be prevented by certification. For this reason, looking at the certificate while buying organic products becomes important and can also have significant effects on the attitudes and behaviors of students (32). Similar to the results of this study, it has been concluded that people who have knowledge about organic products buy more organic products (62–64).

In the study, positive gains were obtained in the attitudes and behaviors of students who consumed organic products. It can be claimed that this situation is caused by the fact that individuals who use organic animal products and develop positive characteristics in themselves with the beneficial properties of this product behave accordingly in their next steps. As the consumption of organic animal products increases, the benefits it provides increase, and the consumer becomes more demanding (32). Similar to the results of this study, it has been found that as the consumption of organic animal products increases, more organic products are consumed (63–65).

The last finding of the study is that there is a weak positive relationship between students’ knowledge and attitudes toward organic animal products. Similarly, there is also a weak positive relationship between students’ attitudes and behaviors toward organic animal products. It is possible to explain this situation with the fact that as the knowledge of organic products increases, attitudes and behaviors will also be affected by this situation. Accurate information about the production stage, health benefits, contributions and returns of organic animal products will lead students to have a certain attitude toward these products and to organize their behaviors accordingly (66, 67). However, the fact that the arithmetic averages of the students in the knowledge dimension are quite low suggests that there are deficiencies in the knowledge dimension. In connection with this deficiency, it is considered as an expected situation that there is a positive but weak relationship between attitudes and behaviors.

The significant differences obtained as a result of the research and the weak relationship between knowledge, attitude and behavior were found. This situation can be evaluated as the increase in the organic product market in Turkey, especially in the last 20 years, has reached the whole society. In addition to the increasing organic product market in Turkey, it is obvious that the health and consumption-related information provided for the consumption of these products also has an effect on students. However, although there are significant developments about organic products, the lack of sufficient and complete information also suggests that the relationship between knowledge, attitude and behavior is not strong enough. In addition to increasing the current information activities for university students staying in dormitories, applications can be implemented regarding the price issue (for example, giving one product as a promotion for buying two organic products), which is the most complained issue of students. In this way, both the consumption of organic products will be increased and the sustainability in organic product consumption will be increased. It was observed that students’ level of knowledge about organic animal products was not sufficient. In this context, students should be given training on organic animal products. Finally, considering the short-and long-term effects of organic animal products and implementing the necessary regulations by the state, it can be recommended to increase access to these products.

## Data Availability

The datasets presented in this study can be found in online repositories. The names of the repository/repositories and accession number(s) can be found in the article/Supplementary material.
