# Supplementary material for: Child development students’ perspectives on organic animal products: knowledge, attitudes and behaviors
Source: Front Nutr. 2025 Jun 25;12:1619260. doi: 10.3389/fnut.2025.1619260 (PMC12239742; doi:10.3389/fnut.2025.1619260)
Supplement: Supplementary file 1 [file Data_Sheet_1.pdf]

First name: Ogukan

Surname: ARAL

Email: ogulcanaral@gmail.com

Organization name: ORSER Control and Certification Ltd. Co.

Addition/removal tick as appropriate

- Addition
- Removal

Individual contributions (as per CRediT Contributor Roles Taxonomy) Only for author additions

Select at least one mandatory contribution:

- ● Writing – original draft
- ● Writing – review & editing

Choose any additional optional contribution as required:

- ● Data curation
- ● Methodology
- Supervision
- ● Conceptualization
- Formal analysis
- Project administration
- ● Validation
- ● Investigation
- Funding acquisition
- Resources
- Visualization
- Software

Reason for change

No changes have been made to the author.

Signature
Only for authors being removed
Authors being added should sign page 9

O. Aral

First name: Yusuf

Surname: CUFADAR

Email: ycufadar@selcuk.edu.tr

Organization name: Selcuk University, Faculty of Agriculture, Department of Animal Science

Addition/removal tick as appropriate

- ● Addition
- Removal

Individual contributions (as per CRediT Contributor Roles Taxonomy) Only for author additions

Select at least one mandatory contribution:

- Writing – original draft
- ● Writing – review & editing

Choose any additional optional contribution as required:

- Data curation
- Methodology
- ● Supervision
- ● Conceptualization
- Formal analysis
- ● Project administration
- Validation
- Investigation
- Funding acquisition
- Resources
- Visualization
- Software

Reason for change

The author has taken an active role particularly in the conceptualization and control of the subject.

Signature
Only for authors being removed
Authors being added should sign page 9

YCufadar

frontiers

First name	Surname	Email
Gül	KADAN	gulkadan@gmail.com

Organization name

Cankırı Karatekin University, Faculty of Health Sciences, Department of Child Development

Addition/removal tick as appropriate

○ Addition ○ Removal

Individual contributions (as per CRediT Contributor Roles Taxonomy) Only for author additions

Select at least one mandatory contribution:

- ● Writing – original draft
- ● Writing – review & editing

Choose any additional optional contribution as required:

- ● Data curation
- ● Methodology
- ○ Supervision
- ○ Conceptualization
- ● Formal analysis
- ○ Project administration
- ● Validation
- ● Investigation
- ○ Funding acquisition
- ○ Resources
- ● Visualization
- ○ Software

Reason for change

No changes have been made to the author.

Signature

Only for authors being removed.
Authors being added should sign page 9.

First name	Surname	Email
Neriman	ARAL	aralneriman@gmail.com

Organization name

Ankara University, Faculty of Health Sciences, Department of Child Development

Addition/removal tick as appropriate

○ Addition ○ Removal

Individual contributions (as per CRediT Contributor Roles Taxonomy) Only for author additions

Select at least one mandatory contribution:

- ○ Writing – original draft
- ● Writing – review & editing

Choose any additional optional contribution as required:

- ○ Data curation
- ● Methodology
- ● Supervision
- ● Conceptualization
- ○ Formal analysis
- ● Project administration
- ○ Validation
- ○ Investigation
- ○ Funding acquisition
- ○ Resources
- ○ Visualization
- ○ Software

Reason for change

No changes have been made to the author

Signature

Only for authors being removed.
Authors being added should sign page 9.

frontiers

First name: Burcin

Surname: Aysu

Email: baysu@aybu.edu.tr

Organization name: Ankara Yildirim Beyazit University, Faculty of Health Sciences, Department of Child Development

Addition/removal tick as appropriate

- [ ] Addition
- [ ] Removal

Individual contributions (as per CRediT Contributor Roles Taxonomy) Only for author additions

Select at least one mandatory contribution:

- [ ] Writing – original draft
- [x] Writing – review & editing

Choose any additional optional contribution as required:

- [x] Data curation
- [x] Methodology
- [ ] Supervision
- [ ] Conceptualization
- [ ] Formal analysis
- [ ] Project administration
- [ ] Validation
- [ ] Investigation
- [ ] Funding acquisition
- [ ] Resources
- [ ] Visualization
- [ ] Software

Reason for change

No changes have been made to the author.

Signature
Only for authors being removed.
Authors being added should sign page 9

BAysu

First name

Surname

Email

Organization name

Addition/removal tick as appropriate

- [ ] Addition
- [ ] Removal

Individual contributions (as per CRediT Contributor Roles Taxonomy) Only for author additions

Select at least one mandatory contribution:

- [ ] Writing – original draft
- [ ] Writing – review & editing

Choose any additional optional contribution as required:

- [ ] Data curation
- [ ] Methodology
- [ ] Supervision
- [ ] Conceptualization
- [ ] Formal analysis
- [ ] Project administration
- [ ] Validation
- [ ] Investigation
- [ ] Funding acquisition
- [ ] Resources
- [ ] Visualization
- [ ] Software

Reason for change

Provide the author list in the order that you would like it to be published and with the correct spelling.

请按照文章发表时您所希望呈现的作者顺序提供相应的作者名单及作者姓名的正确拼写.

	First name(s)	Surname	Email address	Signature ⓘ	Date
01	Oğulcan	ARAL	ogulcanaral@gmailcom	[illegible]	17.06.2025
02	Yusuf	CUFADAR	ycufadar@selcukedutr	[illegible]	17.06.2025
03	Gül	KADAN	gulkadan@gmailcom	[illegible]	17.06.2025
04	Nerlman	ARAL	aralneriman@gmailcom	[illegible]	17.06.2025
05	Burçin	AYSU	baysu@aybu.edutr	[illegible]	17.06.2025
06					
07					
08					
09					
10					
11					
12					
13					
14					
15					
16					
17					
18					
19					
20					
21					
22					
23					
